# Early Changes in Tumor Perfusion from T1-Weighted Dynamic Contrast-Enhanced MRI following Neural Stem Cell-Mediated Therapy of Recurrent High-Grade Glioma Correlate with Overall Survival

**DOI:** 10.1155/2018/5312426

**Published:** 2018-03-14

**Authors:** Prativa Sahoo, Paul Frankel, Julie Ressler, Margarita Gutova, Alexander J. Annala, Behnam Badie, Jana Portnow, Karen S. Aboody, Massimo D'Apuzzo, Russell C. Rockne

**Affiliations:** ^1^Division of Mathematical Oncology, Beckman Research Institute, City of Hope, Duarte, CA, USA; ^2^Division of Biostatistics, Beckman Research Institute, City of Hope, Duarte, CA, USA; ^3^Diagnostic Radiology, City of Hope, Duarte, CA, USA; ^4^Department of Developmental & Stem Cell Biology, Beckman Research Institute, City of Hope, Duarte, CA, USA; ^5^Division of Neurosurgery, City of Hope, Duarte, CA, USA; ^6^Department of Medical Oncology & Therapeutics, City of Hope, Duarte, CA, USA; ^7^Department of Pathology, City of Hope, Duarte, CA, USA

## Abstract

**Background:**

The aim of this study was to correlate T1-weighted dynamic contrast-enhanced MRI- (DCE-MRI-) derived perfusion parameters with overall survival of recurrent high-grade glioma patients who received neural stem cell- (NSC-) mediated enzyme/prodrug gene therapy.

**Methods:**

A total of 12 patients were included in this retrospective study. All patients were enrolled in a first-in-human study (NCT01172964) of NSC-mediated therapy for recurrent high-grade glioma. DCE-MRI data from all patients were collected and analyzed at three time points: MRI#1—day 1 postsurgery/treatment, MRI#2— day 7 ± 3 posttreatment, and MRI#3—one-month follow-up. Plasma volume (*V*_p_), permeability (*K* ^tr^), and leakage (*λ* ^tr^) perfusion parameters were calculated by fitting a pharmacokinetic model to the DCE-MRI data. The contrast-enhancing (CE) volume was measured from the last dynamic phase acquired in the DCE sequence. Perfusion parameters and CE at each MRI time point were recorded along with their relative change between MRI#2 and MRI#3 (Δ_32_). Cox regression was used to analyze patient survival.

**Results:**

At MRI#1 and at MRI#3, none of the parameters showed a significant correlation with overall survival (OS). However, at MRI#2, CE and *λ* ^tr^ were significantly associated with OS (*p* < 0.05). The relative *λ* ^tr^ and *V*_p_ from timepoint 2 to timepoint 3 (Δ_32_ *λ* ^tr^ and Δ_32_ *V*_p_) were each associated with a higher hazard ratio (*p* < 0.05). All parameters were highly correlated, resulting in a multivariate model for OS including only CE at MRI#2 and Δ_32_ *V*_p_, with an *R* ^2^ of 0.89.

**Conclusion:**

The change in perfusion parameter values from 1 week to 1 month following NSC-mediated therapy combined with contrast-enhancing volume may be a useful biomarker to predict overall survival in patients with recurrent high-grade glioma.

## 1. Introduction

High-grade glioma, including glioblastoma (GBM), is the most aggressive type of primary brain tumor. Despite the use of multimodality therapy including surgical resection, chemotherapy, and radiation therapy (RT), the median survival of GBM patients is less than 2 years from diagnosis and only 6 months from recurrence [1]. Previous studies have demonstrated that imaging techniques such as contrast-enhanced magnetic resonance imaging (MRI) can be used in the evaluation of GBM after combined RT and temozolomide (TMZ) therapy (RT-TMZ) [2–4]. With the recent development of cell-mediated therapies, which may induce transient changes in the tumor microenvironment that may be misconstrued as tumor progression, there is an even greater urgency to discover early indicators of response to treatment and correlate these indicators with outcomes including overall survival. T1-weighted dynamic contrast-enhanced MRI (DCE-MRI) sequences are becoming a widely available method for the quantification of perfusion in the brain. DCE-MRI-based perfusion and permeability measures such as the contrast transfer coefficient, blood volume, and blood flow have been proposed as biomarkers for glioma grading [5, 6] and survival prediction following RT-TMZ treatment [2, 7]. Analyzing perfusion parameters derived from DCE-MRI may provide vital information for more accurate assessment of tumor response or progression to a given treatment, including cell-mediated therapies.

Neural stem cells (NSCs), due to their inherent tumor tropism, have the ability to migrate through brain tissue, providing a vehicle to deliver therapeutic agents selectively to invasive glioma foci [8]. NSC-mediated enzyme/prodrug therapies are now being assessed in phase I trials as potential treatment options for patients with GBM [9–12]. Assessing response during or after treatment is critical to optimizing efficacy. NSC targeting of tumor cells may produce alterations in the tumor microenvironment, including changes in blood flow and vascularization. The use of perfusion imaging, DCE-MRI, to assess treatment response of NSC-mediated therapy [10] may have significant clinical impact in evaluating early response to these therapies and has not been previously reported. The objective of this study was to evaluate changes in DCE-MRI-derived perfusion parameters before and after NSC-mediated therapy in patients with recurrent high-grade glioma and to correlate these changes with outcomes as measured by overall survival.

## 2. Patient Selection

Patients were enrolled in a first-in-human pilot safety/feasibility dose escalation clinical trial (NCT01172964) using genetically modified NSCs expressing *E. coli* cytosine deaminase (CD), which converts the prodrug 5-fluorocytosine (5-FC) into the active chemotherapeutic agent 5-fluorouracil (5-FU). Patients were treated on one of the three CD-NSC dose levels: dose 1 (10 × 10^6^ CD-NSCs, 75 mg/kg/day 5-FC), dose 2 (10 × 10^6^ CD-NSCs, 150 mg/kg/day 5-FC), or dose 3 (50 × 10^6^ CD-NSCs, 150 mg/kg/day 5-FC). Details of the treatment schedule, inclusion criteria, and exclusion criteria were previously described [10]. A total of 15 patients ranging from 22 to 63 years old, with a median age of 46, received treatment with CD-NSCs and oral 5-FC. All patients underwent T1-weighted DCE-MRI on a 3T scanner (Verio, Siemens Healthcare, Erlangen, Germany) at three time points: MRI#1 was taken one day after the surgery, during which CD-NSCs were administered along with a catheter placed for additional treatments, followed by a 7-day course of oral 5-FC; MRI#2 was taken 7 ± 3 days after MRI#1; and MRI#3 was taken one month (25 ± 5 days) after MRI#2. MRI scans of all patients were collected and analyzed retrospectively. Three patients were excluded from this study: one patient did not complete the treatment, one patient did not have a one-month follow-up MRI, and one patient's MRI data could not be analyzed due to motion artifact. Therefore, a total 12 patients were included in this analysis. Three patients were treated with dose level 1, 3 patients with dose level 2, and 6 patients with dose level 3. All 12 patients were deceased at the time of data analysis. This study was approved by the local institutional review board.

## 3. Material and Methods

### 3.1. Quantification of Perfusion Parameters

A three-compartment pharmacokinetic leaky tracer kinetic model (LTKM) was fitted to the concentration time curve, and fractional plasma volume (*V*_p_), permeability (*K* ^tr^), and leakage (*λ* ^tr^) were estimated as previously reported [7]. In-house JAVA-based software was used for all DCE computations. Details of the imaging parameters and postprocessing of DCE-MRI data are given in the Supplementary Materials.

### 3.2. Region of Interest (ROI)

As a clinical measurement of lesion size, the last dynamic phase of DCE data was used to identify the lesion on each 2D slice where the lesion was visible. ROIs were drawn manually on each slice, covering the enhancing and nonenhancing components of the whole lesion. Regions of normal vasculature were avoided while drawing these regions of interest. The total number of voxels in the ROI was used as a measure of total contrast enhancement (CE) and of total tumor volume. Only the voxels inside the manual ROI that showed a confidence level of at least 80% in the kinetic model fitting were selected as a mask to exclude voxels with low or noisy contrast enhancement. The mask was then applied to the ROI for both the perfusion and diffusion images. Selecting voxels with the kinetic model includes both early- and late-phase enhancing voxels. For each patient, the mean values for each kinetic parameter for the entire tumor volume were used for statistical analysis.

### 3.3. Comparison of Histology and Imaging

Due to the heterogeneity of imaging and histological findings in high-grade glioma and since it is not possible to know the precise location of a surgical specimen relative to imaging findings, we did not perform a correlative analysis of histology and imaging findings for all patients. Instead, we selected two patients which represented the extreme and opposite MRI perfusion characteristics to demonstrate the consistency of imaging and histological findings. We used the maximum leakage (*λ* ^tr^) and maximum plasma volume (*V*_p_) values within the tumor volume to compare with tissue immunohistochemistry. Presurgical perfusion and diffusion MRI scans were collected for these two cases, and the imaging findings were compared with the histological findings. The surgical tissue specimens were analyzed by a board-certified neuropathologist (MD'A).

### 3.4. Statistical Analysis

Relative change between perfusion parameters at MRI#2 and MRI#3 was calculated as Δ = log(MRI#3/MRI#2) and denoted with Δ_32_, with an analogous convention for the change between MRI#1 and MRI#2. Overall patient survival (OS) was measured from the time of MRI#3 to patient death. Cox regression analysis was performed to correlate MRI imaging parameters *K* ^tr^, *V*_p_, *λ* ^tr^, CE, and ADC and their relative change with OS. We report the hazard ratio, its confidence interval, and the Wald statistic which is the most conservative statistic in this setting. Relative change between perfusion parameters was included in the model so the HR relates to a 100% increase (using the log of the ratio as a measure of relative change), while parametric values were normalized by mean and standard deviation so the HR relates to the change in risk associated with a one-standard deviation increase in the parameter. Evaluating the parameter as a continuous variable, we used the upper tertile as a cut-off for presentation purposes for the key variables, and the statistics associated with those cut-offs are included. Because of the number of variables considered, we chose to include variables in multivariate analysis and Kaplan-Meir survival plots only if the results were statistically significant at *p* ≤ 0.05. One-way analysis of variance (ANOVA) was performed to analyze the variation in the perfusion parameters with respect to the dose level of NSCs. All statistical tests were two-sided, and significance was considered at a value of *p* < 0.05. The statistical software package R, version 3.0.2 (The R Foundation for Statistical Computing, http://www.r-project.org/), was used to perform all statistical analyses.

## 4. Results

### 4.1. Survival Analysis

**Figure 1:**
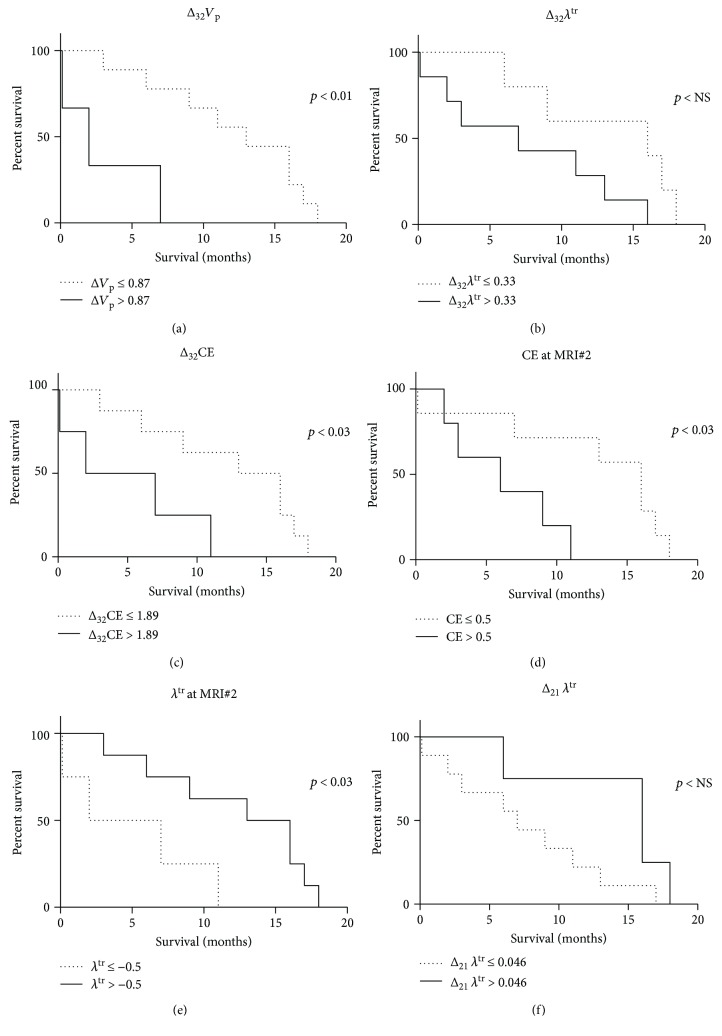
Survival analysis of perfusion kinetic parameters. Kaplan-Meir survival curves indicate that overall patient survival is significantly increased with decreased (a) Δ_32_ *V*_p_, (b) Δ_32_ *λ* ^tr^, (c) Δ_32_CE, and (d) CE at MRI#2. Survival increase is associated with an increase in (e) *λ* ^tr^ at MRI#2 and (f) Δ_21_ *λ* ^tr^.

Cox proportional hazards survival regression results are presented in Table 1. On univariate analysis, none of the parameters showed significant correlation with OS at time points MRI#1 and MRI#3. At MRI#2, the parameters *λ* ^tr^ and CE were significantly associated with OS with *p* < 0.05. The change in the parameter value from MRI#2 to MRI#3 (Δ_32_) for the parameters *λ* ^tr^, *V*_p_, and CE was significantly associated with OS with increased hazard (*p* < 0.05). However, Δ_21_ *λ* ^tr^ and *λ* ^tr^ at MRI#2 were associated with lower hazard (*p* < 0.05). Parameters selected for multivariate analysis were *λ* ^tr^ at MRI#2; CE at MRI#2; Δ_21_ *λ* ^tr^; and Δ_32_ *V*_p_, Δ_32_ *K* ^tr^, and Δ_32_ *λ* ^tr^. ADC did not show any significant correlation with OS at any of the imaging time points. Perfusion parameters and their changes were correlated, resulting in the multivariate model for OS including only CE at MRI#2 and Δ_32_ *V*_p_. Only CE at MRI#2 gives a prediction with *R* ^2^ of 0.26, and when adding Δ_32_ *V*_p_ to the CE at MRI#2, the prediction improves with *R* ^2^ of 0.89. Using the upper tertile, Δ_32_ *V*_p_ (>80% increase) was associated with a median OS of 4.5 months (95% confidence interval (CI): 0.13-NR) versus 14.5 months for Δ_32_ *V*_p_ ≤ 80% increase (CI: 11.0-NR, *p* < 0.05, Wald's test). The upper tertile of CE at MRI#2 (>0.5 SD above the mean) was associated with a median OS of 4.5 months (CI: 2.0-NR) versus 14.5 months for the lower 2/3 of the cases (CI: 9-NR, *p* < 0.05). A combined multivariable model is significant using both Δ_32_ *V*_p_ and CE at MRI#2 as continuous variables or dichotomized with values above their respective cut-offs. The Kaplan-Meier survival curves for Δ_32_ *V*_p_, Δ_32_ *λ* ^tr^, Δ_32_CE, and *λ* ^tr^ at MRI#2; CE at MRI#2; and Δ_21_ *λ* ^tr^ are shown in Figure 1. The survival curves between two groups were significantly different for the parameters Δ_32_ *V*_p_, Δ_32_CE, and *λ* ^tr^ at MRI#2 and CE at MRI#2; however, it was not significant for Δ_32_ *λ* ^tr^ and Δ_21_ *λ* ^tr^.

### 4.2. Survival with respect to Dose Level of NSCs

**Figure 2:**
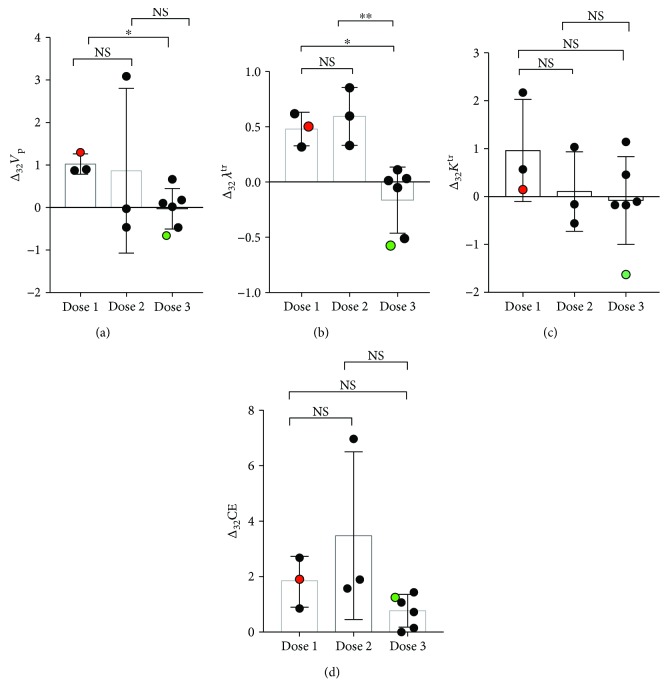
Perfusion kinetics vary with NSC and 5-FC dose level. The relative change in plasma volume (a) Δ_32_ *V*_p_ and leakage (b) Δ_32_ *λ* ^tr^ varies significantly (*p* < 0.05) from dose 1 to dose 2 of NSCs. However, (c) Δ_32_ *K* ^tr^ and (d) Δ_32_CE did not show any significant change with respect to the dose level of NSCs. Patients selected for histological analysis in Figures 3 and 4 are shown in red and green circles, respectively. ^∗^ *p* < 0.05, ^∗∗^ *p* < 0.01.

One-way ANOVA showed that the relative change in plasma volume (Δ_32_ *V*_p_) and leakage (Δ_32_ *λ* ^tr^) significantly decreased (*p* < 0.01) from dose 1 to dose 3 (Figure 2). The mean Δ*V*_p_ values for dose 1 and dose 3 were 1.8 (SD = 0.4, *n* = 3) and 0.07 (SD = 0.2, *n* = 6), respectively. The mean Δ*λ* ^tr^ values for dose 1 and dose 3 were 0.63 (SD = 0.14, *n* = 3) and −0.12 (SD = 0.1, *n* = 6), respectively. No significant difference in CE was found with respect to the dose level of CD-NSC (Figure 2). Overall patient survival also did not show any statistically significant correlation with the dose level of NSC.

### 4.3. Comparison of Imaging and Histological Findings

**Figure 3:**
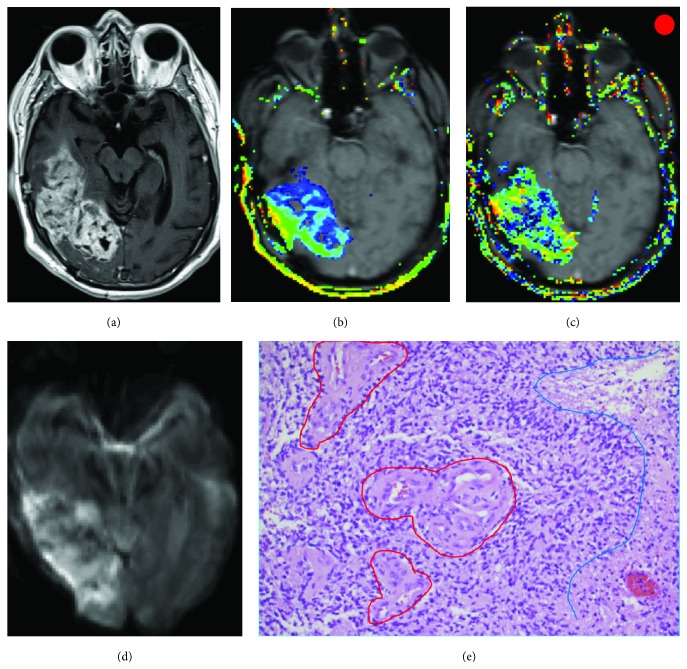
Presurgical images of a 53-year old GBM patient show a heterogonous contrast enhancement on the T1 postcontrast image (a), disrupted blood brain barrier and increased vessel density on leakage *λ* ^tr^ (b) and *V*_p_ (c) maps overlaid on the T1-weighted image, and high cellularity on DWI (d). Histologic specimen (e) shows marked cellularity, frequent microendothelial proliferation (circled in red), and pseudopalisading necrosis (blue line). The patient received CD-NSCs on dose level 1, and the overall survival after NSC treatment was 2 months. The red circle on the top right corner corresponds to the red data points in Figure 2.

**Figure 4:**
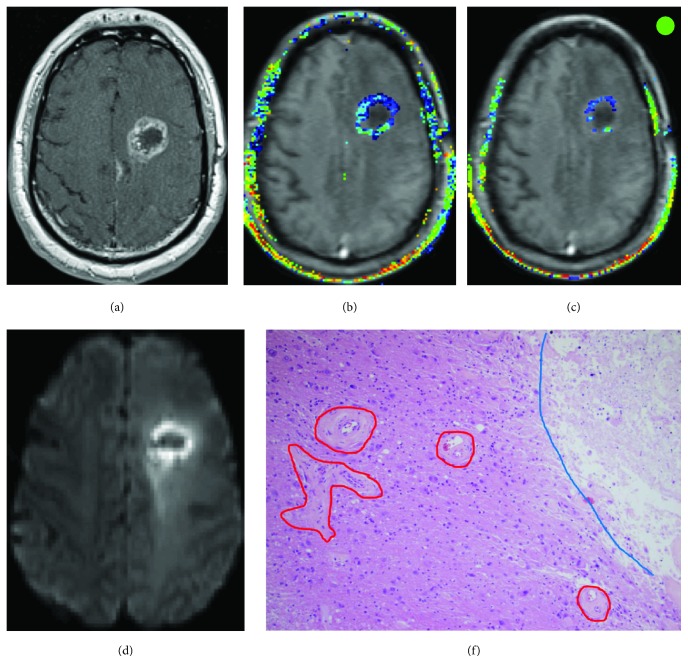
Presurgical images of a 52-year old GBM patient show a ring-enhancing mass with central necrosis on the T1 postcontrast image (a), disrupted blood brain barrier on leakage *λ* ^tr^ (b) and vessel density on *V*_p_ (c) maps overlaid on the T1-weighted image, and cellularity on the diffusion-weighted image (DWI) (d). Histologic specimen (e) shows low-to-moderate cellularity, sclerosed microvasculature (circled in red), and hyalinizing necrosis (blue line). The patient received CD-NSCs on dose level 3, and the overall survival after NSC treatment was 18 months. The green circle on the top right corner corresponds to the green data points in Figure 2.

The imaging findings of perfusion and cellularity were consistent with the gross histological findings for the two patients selected to represent the high and low range of perfusion characteristics in this patient population (Figure 2; case 1 (red) and case 2 (green)). In case 1, imaging showed heterogeneous contrast enhancement with increased perfusion (increased *V*_p_ and *λ* ^tr^). The haemotoxylin and eosin (H&E) preparations of the specimen of case 1 showed a markedly cellular recurrent GBM, displaying increased mitotic activity and pseudopalisading necrosis. Numerous newly formed glomeruloid vessels with characteristic features of microendothelial proliferation were frequently seen (Figure 3). In contrast, case 2 showed a moderate ring enhancement with central necrosis in a T1-weighted postcontrast image. DCE-MRI-derived parameters showed moderate leakage and slightly increased plasma volume (*V*_p_). The H&E preparations of the specimen of case 2 showed low-to-moderate cellularity with areas of hyalinizing necrosis, devoid of nuclear pseudopalisading (Figure 4). The microvasculature was predominantly devoid of proliferative endothelium and frequently surrounded by sclerosis.

## 5. Discussion

We analyzed the T1-weighted DCE-MRI perfusion parameters (*K* ^tr^, *V*_p_, and *λ* ^tr^) and contrast enhancement measured at initiation and completion of NSC + 5-FC treatment and at one-month follow-up to evaluate changes in perfusion parameters and their correlation with overall survival of patients with recurrent high-grade glioma. In addition, we explored the variability in perfusion parameter values with respect to the NSC dose level. The change in *V*_p_, *λ* ^tr^, and CE between the end of treatment and follow-up at one month showed significant negative correlation with overall survival while the change in *K* ^tr^ and ADC did not. At the end of treatment (MRI#2), the leakage parameter *λ* ^tr^ showed a positive correlation with OS. There was a significant change in the relative change in plasma volume (Δ_32_ *V*_p_) and leakage (Δ_32_ *λ* ^tr^) observed between dose 1 and dose 3, suggesting a dose-dependent effect on the changes in perfusion in the tumor microenvironment associated with the NSC-mediated therapy.

Response to treatment for high-grade glioma is generally evaluated on MR images using the Response Assessment in Neuro-Oncology (RANO) criteria in which change in tumor size on postcontrast images is a major component. However, increased tumor size in a contrast-enhanced T1-weighted image does not always reflect true progression. Pseudoprogression could be related to postsurgical changes, treatment-induced inflammation, and ischemia [16]. These factors might be the reasons why contrast-enhancing volume alone showed the lowest hazard ratio as compared to other perfusion parameters with *R* ^2^ = 0.26 at the end of treatment. When adding the relative change in plasma volume (Δ_32_ *V*_p_) to the CE at MRI#2, the prediction improves with *R* ^2^ = 0.89. Several studies have focused on quantitative imaging sequences including DCE-MRI, to assist clinicians in evaluating response to treatment more accurately [2–4, 17, 18].

Histologically, progressive high-grade glioma involves neoangiogenesis, resulting in high permeability and disrupted blood brain barrier (BBB). Several studies have demonstrated that relative cerebral blood volume (rCBV) (a perfusion parameter derived from DCE-MRI) correlates with tumor aggressiveness, microvessel density, and patient survival in GBM patients [2, 19]. Disrupted BBB leads to the increase in contrast material leakage into the extravascular extracellular space (EES), with a subsequent increased image contrast which results in overestimation or underestimation of rCBV with DCE-MRI. For this reason, leakage correction in rCBV quantification has been investigated and shown to provide a more reliable estimation of blood volume [19, 20]; therefore, we use the kinetic parameter *V*_p_ as a measure of blood volume in this study. In general, *V*_p_ is defined as the fractional plasma volume; however, *V*_p_ can be converted into blood volume by multiplying it with the proportionality constant (*ρ*/*H*), where *ρ* is the assumed brain tissue density (1.05 g/ml) and *H* = (1 − *H*_art_)/(1 − *H*_cap_) to account for the difference in hematocrit between capillaries and large vessels (in this study, *H*_cap_ = 0.25 and *H*_art_ = 0.45) [21].

In our study, all MRI data were postoperative. We did not find any significant correlation between the perfusion parameters and patient survival at the postoperative, pre-5-FC time point (MRI#1). This is likely because there is very little residual tumor or contrast enhancement immediately following surgery. Interestingly, leakage *λ* ^tr^ and CE showed a significant (*p* < 0.05) correlation with overall survival for MRI#2, with CE negatively correlated and *λ* ^tr^ positively correlated with OS. Although this is a small retrospective study, these findings suggest that a higher mean leakage in the tumor volume at the beginning of treatment may predict a positive response to the treatment. Because the BBB restricts the ability of drugs to reach their site of action, a larger leakage could facilitate the perfusion of enzyme-activating drugs through the tumor resulting in improved treatment efficacy. ADC has been shown to be inversely related to cell density in tumor tissue. Homing of NSCs in cancer tissue may therefore decrease ADC, while delivery of chemo can cause cell death resulting in increased ADC. The combination of these effects may explain why ADC did not show significant correlation with overall survival.

To ground our imaging findings with histological features, we selected two patients that represented extreme and opposite perfusion characteristics. Due to the inability to precisely determine the origin of the tissue specimen to regions in the imaging, we qualitatively compared the imaging findings to the gross histological features of cell density, vascular proliferation, microvasculature, and necrosis and found the imaging-derived estimates to be consistent with the histologic findings. In both cases, the imaging findings were consistent with the gross histologic features found in the tissue specimens collected at the time of surgery, prior to NSC therapy.

NSC dose may affect perfusion parameters; however, in the present study, the patient cohort at the individual dose level is very small for any conclusive result and the dose levels used in this study are a combination of NSC dose and 5-FC dose. Changes in perfusion could be related to homing of NSCs to the tumor site resulting in normalized/repaired tumor vessels, can be the result of the therapeutic antivascular effects of 5-FU, or both. Additionally, the surgical manipulation would likely impact the measured perfusion parameters. We cannot rule out the possibility that the surgical manipulations that correlated with the best clinical outcome and ability to maximize the activity of the NSCs were reflected in the perfusion parameters, resulting in the association of the perfusion parameters with overall survival. Moreover, changes in perfusion can also depend upon tumor type, localization, NSC survival, homing and inhibition, delivery of 5-FC, and activation of 5-FU. Understanding the effect of NSCs and antivascular effects of 5-FU individually on perfusion parameters requires further investigation.

In conclusion, our study shows the potential value of inclusion of perfusion parameters, including relative change in plasma volume (Δ_32_ *V*_p_) and leakage (Δ_32_λ^tr^) along with conventional contrast volume as biologically meaningful early surrogate endpoints and as early indicators of patient survival after NSC-mediated therapy in patients with recurrent high-grade gliomas. A decrease in Δ_32_ *V*_p_ and Δ_32_λ^tr^ may be an early indicator of improved patient survival, while the contrast-enhanced MRI-based size criteria may not be sufficient in this setting. Future studies are required to investigate the surgical and dose-dependent effects of NSCs on perfusion kinetics and ultimately on treatment response and overall survival. Additional confirmatory studies are warranted.

## Figures and Tables

**Table 1:** Cox proportional hazards survival regression results of perfusion parameters and apparent diffusion coefficient (ADC) as continuous measures at all time points MRI#1, MRI#2, and MRI#3. Δ represents the relative change. Δ_32_ = ln(MRI#3/MRI#2), and Δ_21_ = ln(MRI#2/MRI#1).

Parameters	Statistics	MRI#1	MRI#2	MRI#3	Δ_21_	Δ_32_
*V*_p_	*p* value					<0.01^∗^
	HR	NS	NS	NS	NS	20.67
	CI					[2.5,170.1]

*λ* ^tr^	*p* value		<0.05^∗^		<0.05^∗^	<0.01^∗^
	HR	NS	0.47	NS	0.17	17.08
	CI		[0.23, 0.98]		[0.03, 0.9]	[1.71, 171]

*K* ^trans^	*p* value					
	HR	NS	NS	NS	NS	NS
	CI					

CE	*p* value		<0.05^∗^			<0.05^∗^
	HR	NS	2.13	NS	NS	2.02
	CI		[1.01, 4.5]			[1.03, 3.98]

ADC	*p* value					
	HR	NS	NS	NS	NS	NS
	CI					

^∗^Statistically significant. HR: hazard ratio; CI: 95% confidence interval; NS: not significant. *p* value for Wald's test.

## Supplementary Materials

Supplementary Materials Included in the supplementary materials are the MRI scanning protocol and quantification of perfusion parameters.
