# Supplementary material for: Early Changes in Tumor Perfusion from T1-Weighted Dynamic Contrast-Enhanced MRI following Neural Stem Cell-Mediated Therapy of Recurrent High-Grade Glioma Correlate with Overall Survival
Source: Stem Cells Int. 2018 Mar 14;2018:5312426. doi: 10.1155/2018/5312426 (PMC5872616; doi:10.1155/2018/5312426)
Supplement: Supplementary Materials — Included in the supplementary materials are the MRI scanning protocol and quantification of perfusion parameters. [file 5312426.f1.pdf]

Early changes in tumor perfusion from dynamic contrast enhanced T1-weighted MRI following neural stem cell-mediated therapy of recurrent high grade glioma correlates with overall survival

SUPPLEMENTAL MATERIALS AND METHODS

MRI Scanning protocol

All patients underwent the same DCE-MRI scanning protocol as follows: repetition time/echo time (TR/TE):9.3/4.29ms; flip angle:15°; acquisition matrix 81 × 192; field of view (FOV) 192 × 280 (mm^2); slice thickness 5mm; number of slices 16; number of dynamic phase 50; temporal resolution 5s. At the fifth time point of the DCE-MRI data acquisition, 0.2 mmol/kg body weight of Gd-BOPTA (Multihance, Bracco, Italy) was administered intravenously with a power injector at a rate of 3.5 ml/s, followed by a bolus injection of a 30ml saline flush. Three different flip angle (2°, 5° and 10°) pre-contrast T1-weighted images were acquired for absolute quantification of T_1 relaxation time. The total acquisition time for dynamic scan was 4min 10sec.

Quantification of Perfusion Parameters

Pre-contrast T1 was measured by using pre-contrast T1-weighted images acquired at three different flip angles [1]. Contrast agent concentration time curves were computed voxel-wise using Signal intensity time DCE-MRI data and pre-contrast T1. Automatic arterial input function was extracted by using the algorithm described in [2]. A three-compartment pharmacokinetic leaky tracer kinetic model (LTKM) was fitted to the concentration time curve and fractional plasma volume (V_p), permeability (K^{trans}), leakage (λ^{tr}) were estimated as previously reported [3]. An in-house JAVA-based software was used for all DCE computations.

References

1. Liberman G, Louzoun Y, Ben Bashat D. T_1 Mapping using variable flip angle SPGR data with flip angle correction. *J Magn Reson Imaging*. 2014;40(1):171-180. doi:10.1002/jmri.24373.
2. Singh A, Rathore RKS, Haris M, Verma SK, Husain N, Gupta RK. Improved bolus arrival time and arterial input function estimation for tracer kinetic analysis in DCE-MRI. *J Magn Reson Imaging*. 2009;29:166-76.
3. Sahoo P, Rathore RKS, Awasthi R, et al. Subcompartmentalization of extracellular extravascular space (EES) into permeability and leaky space with local arterial input function (AIF) results in improved discrimination between high- and low-grade glioma using dynamic contrast-enhanced (DCE) MRI. *J Magn Reson Imaging*. 2013;38(3):677-688.
